# Phosphorelay changes and plasticity underlie the life history evolution of Bacillus subtilis sporulation and germination in serial batch culture

**DOI:** 10.1099/mic.0.001540

**Published:** 2025-03-17

**Authors:** Kathleen A. Sagarin, Elizabeth Ouanemalay, Hilda Asante-Nyame, Vera Hong, Chloe De Palo, Frederick M. Cohan

**Affiliations:** 1 Department of Biology, Wesleyan University, Middletown, CT, USA

**Keywords:** adaptive divergence, bet-hedging, germination efficiency, parallel evolution, spore loss

## Abstract

Bacterial endospores facilitate survival in extreme and unpredictably fluctuating environments. However, under abundant nutrient conditions, the production of endospores is quickly reduced or lost. We hypothesized that endospore-forming bacteria exposed to frequent cycling of nutrient availability would evolve reduced sporulation efficiency. We employed replicated batch culture for 11 transfers to test the effects of rapid nutrient cycles on the evolution of the life history traits of sporulation, germination and growth in *Bacillus subtilis*. We periodically measured total cell and endospore densities during the period between transfers. Replicates evolved in parallel behaviourally and genetically. By the fourth transfer, we saw a reduction in endospore production, which continued to decline throughout the experiment. Our results support a decreased likelihood of sporulation being driven by frequent nutrient renewal. The proportion of endospores germinating after transfer increased significantly by the end of the experiment through the effects of plasticity alone. Every evolved replicate culture displayed colony dimorphism: the dominant morphology being translucent with reduced sporulation ability and the rarer being opaque with accelerated sporulation and highly efficient germination. Colony dimorphism was reflected in the genomes, with all isolates with reduced sporulation having mutations in elements of the sporulation phosphorelay, particularly *kinA*. Some opaque colonies had no mutations, indicating that those adaptive changes occurred through plasticity. These results suggest that our selection conditions of nutrient cycling resulted in the parallel evolution of communities of ecologically diverse strains, where most reduced sporulation while a smaller proportion accelerated it.

**Impact Statement**

This project is the first to examine the evolutionary response of sporulation dynamics in *Bacillus subtilis* to predictably fluctuating nutrient conditions. Specifically, this project measured spore density at multiple time points during growth and stationary phase, allowing the estimation of germination proportion and rates of change of total cell and endospore densities. This work is of interest to the fields of microbiology, evolution, ecology and genetics. We explore an environmental condition that is intermediate between driving the total loss of sporulation and stably selecting for sporulation. Major findings include the first *in vitro* observation of population-wide tuning of sporulation efficiency without the loss of sporulation ability. We also found the parallel emergence of two distinct colony morphotypes corresponding to higher and lower sporulation efficiency. This is the first recorded instance of sporulating strains improving sporulation under relaxed selection and co-existing with lower-sporulating mutants, indicating that our culture environment was not simply driving sporulation loss. The colony morphotypes broadly fell into genetic categories as well, with multiple independent mutations in phosphorelay elements in isolates with lower sporulation efficiency, particularly in *kinA*, and the persistence of the ancestral genome in those with higher sporulation efficiency. The latter finding is particularly interesting, indicating dramatic changes in sporulation behaviour through plasticity alone. Our results align with other studies showing loss or reduction in sporulation and provide further evidence that sporulation is tunable to the environment.

## Introduction

Many bacteria of the phylum *Bacillota* can survive harsh conditions for a long time by undergoing sporulation to form durable, dormant endospores, which can later germinate into a vegetative cell [1,3]. While the ability to sporulate is a widespread and broadly conserved trait within *Bacillota*, there is substantial physiological and evolutionary variety and changeability in the sporulation process and consequent endospores across multiple taxonomic levels [4,17]. This lability generally represents adaptability to harsher or milder environments, often related to nutrient conditions [6,7, 11, 15, 18, 19].

Sporulation is a response to nutrient deprivation or crowding, during which a subset of the population forms endospores, while the rest remain vegetative [12,20,22]. This stochasticity in sporulation is a form of bet-hedging [23,24]. Vegetative cells have a short-term fitness advantage because they can quickly exploit nutrients, should they become available, and endospores have an advantage if nutrients remain sparse [21,23, 24]. Sporulation is reversible up to a point, and cells that delay committing to it are in a better position to exploit renewed nutrient availability than those cells that sporulate early [12,14, 21, 25]. Moreover, in nature, endospores that delay germination until after nutrient availability recurs (termed hyperdormant) may do so under more favourable conditions. Endospores that delay germination also constitute a ‘spore bank’, like a plant’s seed bank. These characteristics of germination are also bet-hedging strategies [16,26, 27].

When endospore-forming bacteria evolve under nutrient-rich or milder conditions, vegetative cells are favoured and sporulation is reduced or lost. Mild conditions underlie sporulation loss in major clades and natural populations within *Bacillota* [6,28]. Experimental evolution in which endospore-forming bacteria are grown in continuous culture, such as in a chemostat, results in the rapid loss of the ability to sporulate [7,19]. Frequent passage to fresh nutrients, on agar plates or in liquid culture, also leads to the loss of sporulation ability [11,29]. On the other hand, long periods of starvation lead to increased production of spores [18]. An intermediate environmental condition, between driving loss of sporulation and retention or improvement of it, has yet to be explicitly defined.

An intermediate environmental condition might result in an intermediate sporulation response because sporulation ability is not as simple as present or absent. Sporulation efficiency, the proportion of bacteria in a population that complete sporulation *sensu* Maughan and Nicholson [10], is tunable to the environment. Studies focusing on the binary of sporogenous (sporulating) and asporogenous (non-sporulating) have not recorded the efficiency of sporulation in sporogenous strains [7,11, 19]. Furthermore, mutants with reduced sporulation efficiency (oligosporogeny) may even arise more frequently than completely non-sporulating (asporogenous) mutants in *Bacillus subtilis* [9,30]. Oligosporogeny can occur because sporulation initiation has multiple checks and balances, each of which can be altered, thus changing the threshold of sensitivity [13,20, 22, 31,33]. Oligosporogeny-causing mutations can also occur in metabolism genes, genes encoding proteins involved in spore formation, and other functions peripheral to sporulation initiation [34,35].

The goal of this study was to record the behavioural and genetic response of *B. subtilis* to a selection regime that imposed intermittent nutrient availability, when phases of nutrient abundance drive loss of sporulation ability and phases of starvation drive improved sporulation. We employed serial batch culture, which imposes a predictably fluctuating nutrient cycle, or ‘seasonality,’ where nutrients are highest after transfer and decline until the next transfer, creating a short period of nutrient deprivation [36,38]. We used a 56-h batch length, giving our populations at least 32 h in the stationary phase, which is sufficient time to complete sporulation to the point of wet heat resistance before each transfer [10,39]. We expected that we would see oligosporogeny, since preliminary testing (unpub.) showed no evidence of complete starvation by 56 h, but we were surprised to find that a small percentage of strains retained and, by some measures, improved their sporulation. The genetic response, as measured through whole-genome sequencing of individual isolates, was strongly parallel across the oligosporogenous strains, with all of them having mutations affecting the sporulation initiation phosphorelay. Surprisingly, the strains that retained and improved their sporulation were not genetically different from the ancestor, indicating adaptation through plasticity. In contrast to complete sporulation loss that occurs under continuous culture conditions and improvement of sporulation under long starvation, our experimental evolution resulted in a range of degrees of oligosporogeny driven by changes in the phosphorelay [7,19]. The co-occurrence of oligosporogeny with the plastically changed ancestor indicated that multiple adaptive behaviours arose under our culture conditions.

## Methods

### Strain

We conducted our experiments with *B. subtilis* BKK28260 (*trpC2 ∆leuC::kan*), a derivative of *B. subtilis* strain 168, which we obtained from the *Bacillus* Genetic Stock Center in Columbus, OH [35].

### Experimental evolution

To create our microcosms, we used a single colony of BKK28260 on a lysogeny broth (LB) agar plate (LB Agar, Miller, Fisher Scientific catalogue no. BP1425) to inoculate an overnight liquid culture of Schaeffer’s sporulation medium (SSM [10], with 0.5 µg ml^−1^ kanamycin [40]. SSM is the medium known for best promoting sporulation in *B. subtilis* [41]. We cultured the bacteria in 15 ml of SSM in a vertical Falcon^®^ 50 ml polypropylene conical tube (Corning, product no. 352098). We incubated all liquid cultures at 37±1 °C and 150 r.p.m. orbital shaking. We used the overnight culture to inoculate a 56-h culture at a 1:1,000 dilution, which we called transfer 0 (T0). We established four evolutionary microcosms from 1:1,000 dilutions of the 56-h culture of BKK28260 into fresh SSM, which we called transfer 1 (T1). We transferred evolutionary microcosms at a 1:1,000 dilution in serial batch culture every 56 h for a total of 11 transfers (not including T0), or ~110 generations. We froze samples of the overnight culture of BKK28260 at −80 °C in 25% glycerol (representing T0), as well as samples of all replicate cultures at the time of transfer at −80 °C in 25% glycerol at transfers T4, T10 and T11.

### Isolates

**Fig. 1.**
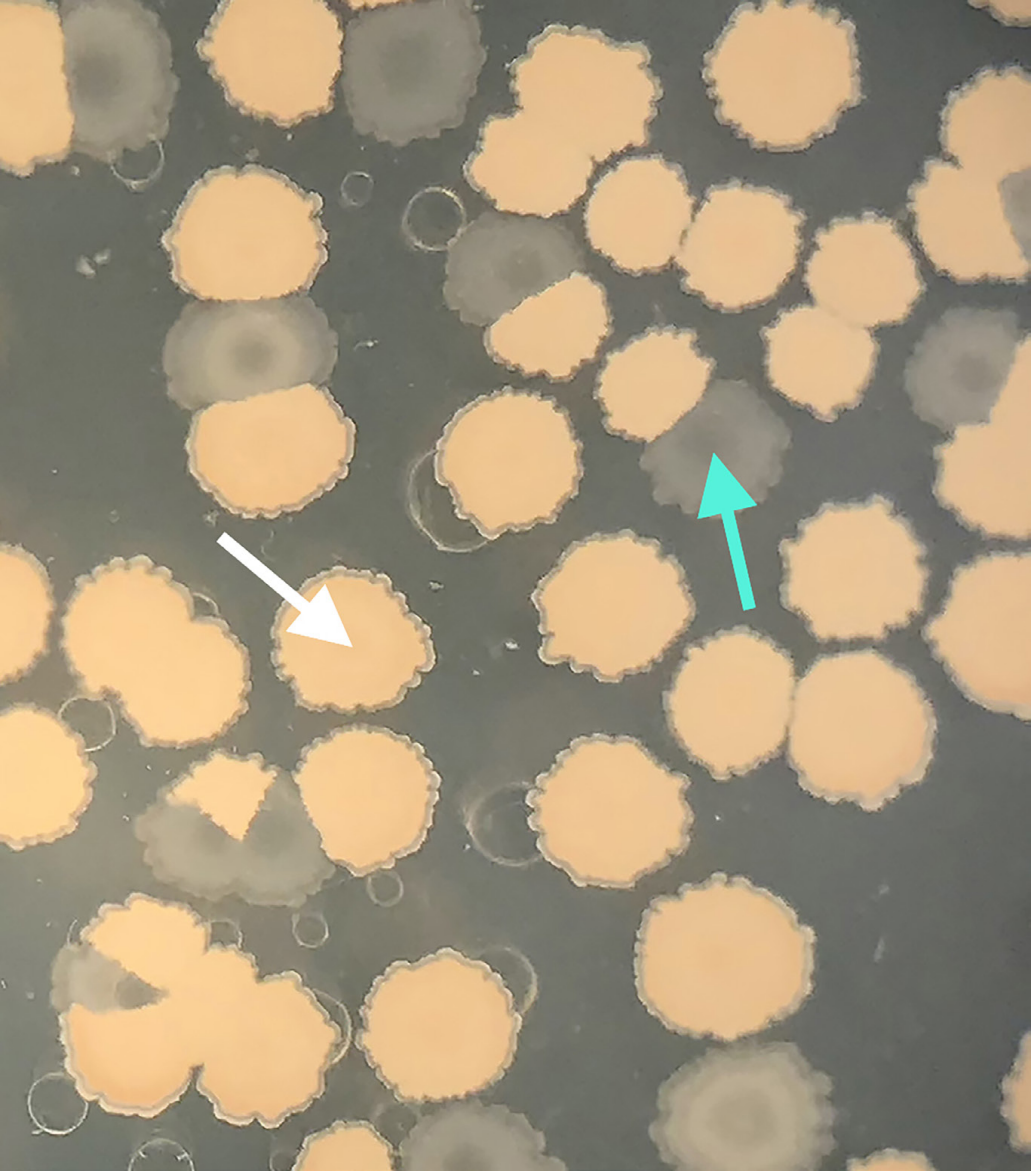
Colony morphology variation in an evolved population. Colonies were plated 12 h after the fourth transfer and were incubated for 3 days at 37 °C. White arrow: a colony that is sporogenous and brown, the condition of the ancestor. Cyan arrow: a colony that is translucent, a derived condition; similar colonies were confirmed to be oligosporogenous. White empty rings are water droplets on the lid of the agar plate.

To obtain isolates at the end of the selection regimen, we used samples frozen from evolutionary replicates A–C at transfer 11 to inoculate SSM overnight cultures. We diluted the overnight cultures to ~10^−5^ and plated onto LB agar plates before and after heating at 80 °C for 10 min to kill vegetative cells. After ~48 h of incubation at 37 °C, we streaked individual colonies for isolation onto separate LB agar plates. Because the 56-h nutrient-cycling selection resulted in two distinct colony morphologies, either opaque and brown (putatively sporogenous) or white and translucent (putatively asporogenous) (Fig. 1) [1], we selected isolates randomly from among the colonies of each morphology, to ensure sampling included the colony morphology differences. We then vortexed individual colonies from the isolate plates in 500 µl of SSM, added 500 µl of sterile 50% glycerol and then split into two 2-ml Eppendorf tubes before freezing at −80 °C. Isolates were named in the following style: r (relaxed selection, *sensu* Maughan *et al*. [42]), A–D (the evolutionary replicate of origin), R/O (plated from a sample p**r**evious to or p**o**st-heating, respectively), T/O (translucent or opaque colony presentation, respectively) and a number to distinguish isolates with the same origin and colony presentation [42].

### Quantifying growth, germination, sporulation and lag time

We initially measured growth and sporulation/germination curves at transfers T1, T4, T7 and T10 in the following manner. We determined total cell density (vegetative cells and endospores) by plating unheated culture samples and endospore density by plating culture samples heated at 80 °C for 10 min to kill vegetative cells. We measured total cell densities and endospore densities at the time of transfer in the old culture (0 h) and specific time points after transfer into fresh medium (for evolutionary microcosms: at 8, 12, 16, 24, 36 and 56 h; for isolates: at 3, 6, 12 or 14, 24, 36 and 56 h). Due to discrepancies resulting from multiple researchers taking measurements, we unfroze samples from the initial overnight culture of the ancestor and samples from transfer 10 cultures and grew them in SSM for 56 h, and then, a single researcher measured the densities at 0, 3, 6, 14, 24, 36 and 56 h after transfer, giving a T1 and a T11 measurement, respectively.

For the evolved isolates, we determined growth, sporulation and germination curves in the following manner. First, we streaked frozen samples of the isolates onto LB agar plates and incubated the plates at 37 °C for ~24 h. Then, we inoculated a single colony into SSM and incubated for 56 h at 37 °C and 150 r.p.m. After those 56 h, we transferred the cultures to three replicates of fresh SSM and measured total cell and endospore densities in each replicate across the following 56-h period.

We defined sporulation efficiency as the percentage of the total cell population in the form of endospores at 56 h. We calculated germination efficiency as 1 − (lowest endospore density between 3 and 14 h/endospore density at 0 h), giving the proportion of the population that germinated. We estimated lag time by calculating the intercept of the regression line of log-transformed total cell density against time during exponential growth with the starting log-transformed total cell density.

### Statistics and data visualization

We used spline regression analysis to compare the first and last transfer microcosm-level time series data and the ratio of endospores to total cell density over time. We used the R packages stats and lspline (R v. 4.2.2, RStudio v. 2023.3.0.386) [43,45]. We used the lm function to make a linear regression model with log_10_(cell density) as our dependent variable, and transfer by time as our interacting independent variable, with spline breaks at 6 and 24 h. We chose our spline breaks considering 0–6 h to be early growth and germination, 6–24-h late growth and sporulation and 24–56-h stationary phase. For endospore density, we used the same model with the addition of replicate as a random variable, as replicate D had significantly different endospore density from 0 to 12 h. For the ratio of endospores over time, we used log_10_(endospore density/total cell density) as the dependent variable. For the significance threshold in all tests, we used 0.05 as the baseline *α* with a Holm–Bonferroni correction [46,48]. We calculated germination proportion as 1 – (smallest endospore density/starting endospore density) per replicate and then performed a two-tailed paired Student’s t-test comparing germination proportion at the beginning and end of the experiment. We compared sporulation efficiency at the first transfer to the last transfer with two-tailed Student’s t-tests. We took 0-h comparisons from the spline regression.

We also used spline regressions to compare the time series of isolate strains from the evolved populations to the ancestral time series, with the same model as the population-level regression using strain as an independent variable instead of transfer. For comparisons between translucent and opaque isolates, we used two-tailed Welch’s t-tests.

We made time series graphs in R using the packages compilation tidyverse and package cowplot [49,50]. We performed and visualized the principal component analysis (PCA) in R using the in-built prcomp function to generate the correlation matrix and ggplot2 from the tidyverse package to create the graph. For the PCA dataset, we used all combinations of time point and total cell or endospore density.

### Genome sequencing

We sent samples of isolates and the ancestral strain (BKK28260) to AmpSeq (Gaithersburg, MD, USA) for DNA extraction with the Zymo Microbiome Kit, library prep with NEB Ultra II FS and sequencing at a minimum of 100× depth with forward and reverse primers on the AVITI sequencer from Element Biosciences. AmpSeq removed adapters from reads prior to making the fastq.gz files available.

### Genome analysis

We uploaded our raw read fastq.gz files to the Galaxy web platform and quality filtered with FASTP [51,52]. We mapped the filtered reads to the published *B. subtilis* 168 reference genome (NCBI RefSeq: GCF_000009045.1, GenBank: GCA_000009045.1) via Bowtie2 [53]. From the Bowtie2 alignments, we identified indels and SNPs using bcftools mpileup, bcftools call with consensus calling, bcftools filter (with QUAL>20 and DP>4) and bcftools query to extract a file containing the indels and SNPs [54]. The ancestral strain, BKK28260, had 42 SNPs and 8 insertions compared to the published *B. subtilis* 168 genome. We labelled insertions and SNPs shared among the ancestor and all isolates as the ancestral condition and removed them. We treated homopolymer indels in homopolymers of three or more as errors from sequencing and ignored them. The raw read fastq files are available in the National Center for Biotechnology Information Sequence Read Archive under the BioProject accession number PRJNA1172268.

### Estimating impacts of mutations

We estimated the projected impacts of the SNPs and indels on protein function using SnpEff [55]. The hypothetical secondary structures of mutated rRNAs in our isolates were predicted using the RNAfold WebServer with default settings, which uses the Turner 2004 [56] model of energy parameters [56,58]. The ancestral (BKK28260) strain’s sequences of *rrnI-16S* and *rrnH-23S* were also predicted this way to be used for comparison to the mutated rRNAs in the isolates, as those sequences in BKK28260 diverged from the published *B. subtilis* 168 sequences. All our rRNA sequences had different predicted minimum free energy and centroid secondary structures, so we used comparative minimum free energies as a proxy for the impact of the mutations (Table 1).

**Table 1.** Complete account of genetic changes in evolved oligosporogenous isolates as compared to the ancestor genome. Position is as compared to the published *B. subtilis* 168 reference genome. BKK score refers to the average sporulation proportion recorded in Koo *et al*. [35] when the specified gene was deleted, and NA means the gene was not deleted and therefore not scored. *kinA* is bolded to highlight its independent mutations in multiple isolates. Estimated impact for non-rRNA genes refers to the impact on protein function, and for rRNA genes, it is the difference in estimated minimum free energy in kilocalories per mole of the predicted rRNA secondary structures compared to the ancestor, where MFE is the minimum free energy prediction and Cen is the centroid prediction. Sporogenous isolates are not included as they had no genetic differences from the ancestor

Isolate	Gene	Position	Type	BKK score	Estimated impact	Gene description
rART1	***kinA***	1471559	SNP; missense	0.09	Moderate	Sporulation-specific ATP-dependent protein histidine kinase, involved in the phosphorelay
rART1	*ydfQ*	599017	SNP; stop gained	0.9	High	Putative thioredoxin or thiol-disulphide isomerase
rART1	*yerC*	716660+	Indels; frameshifts and in-frame insertions	0.85	High to moderate	Transcriptional repressor of histidine operons
rBRO1	***kinA***	1470041	SNP; stop gained	0.09	High	Sporulation-specific ATP-dependent protein histidine kinase, involved in the phosphorelay
rBRO1	*gtaB*	3666234	SNP; synonymous	0.59	Low	UTP-glucose-1-phosphate uridylyltransferase
rBRO1	*rrnI-16S*	161668	SNP	na	MFE: −3.3, Cen: −12	16S ribosomal RNA
rBRT1	***kinA***	1471691	SNP; missense	0.09	Moderate	Sporulation-specific ATP-dependent protein histidine kinase, involved in the phosphorelay
rBRT1	*rrnI-16S*	161239, 161449	Reversions	na	MFE: +0.7, Cen: +0.1	16S ribosomal RNA
rBRT1	*rrnH-23S*	169838, 170390	Reversions	na	MFE: +3.6, Cen: +80.9	23S ribosomal RNA
rBRT1	*rrnG-16S*	171679	Reversion	na	MFE: +0.9, Cen: +0.9	16S ribosomal RNA
rCOT1	*rapA*	1316188	SNP; missense	1.37	Moderate	Response regulator aspartate phosphatase, involved in the phosphorelay
rCOT1	*infA*	147610	SNP; missense	na	Moderate	Initiation factor IF-I
rCOT1	*rrnH-23S*	169384, 169385, 169875	SNPs	na	MFE: −0.5, Cen: −13.1	23S ribosomal RNA
rCRT2	***kinA***	1471217	SNP; missense	0.09	Moderate	Sporulation-specific ATP-dependent protein histidine kinase, involved in the phosphorelay
rCRT2	*rrnI-16S*	161668	SNP	na	MFE: −3.3, Cen: −12	16S ribosomal RNA
rCRT2	*rrnG-16S*	171679	reversion	na	MFE: +0.9, Cen: +0.9	16S ribosomal RNA

## Results

### Changes in culture dynamics under 56-h nutrient-cycling selection

**Fig. 2.**
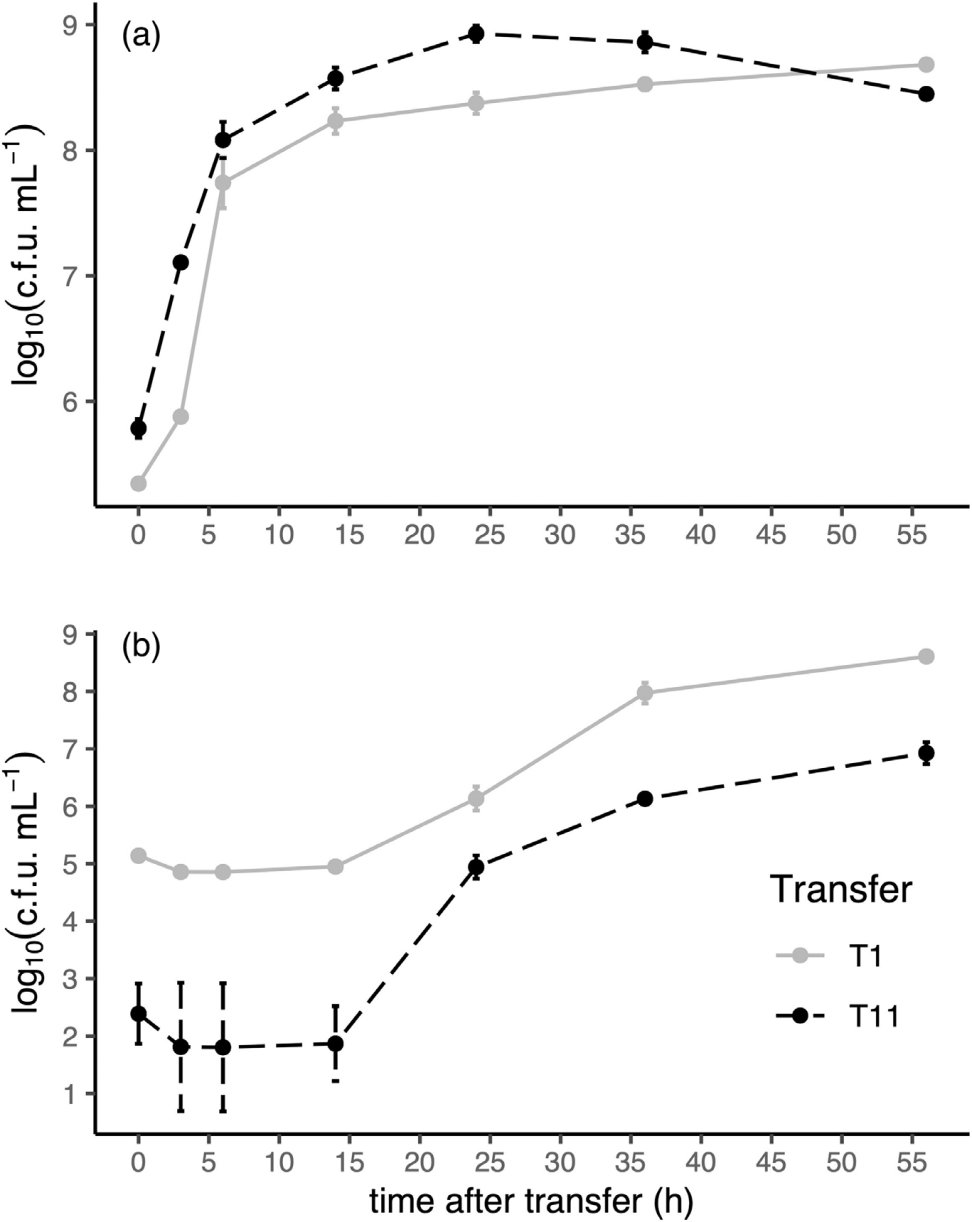
Comparison of the ancestral and evolved population total cell and endospore dynamics over 56 h of culturing. (a) Total cell dynamics, measured from unheated samples, therefore including the pool of vegetative cells and endospores. (b) Endospore dynamics, measured from heated samples, therefore including only endospores. Grey solid line: ancestral condition, recorded after the first transfer. Black dotted line: evolved condition, recorded after the eleventh transfer. c.f.u. ml^−1^: c.f.u. per millilitre, a measure of cell density. Log is log_10_. Vertical bars represent sd, which are smaller than the point if not visible. *N*=4 evolutionary replicates per transfer.

We statistically compared the total cell and spore density measurements taken during the first transfer (T1) to those from the last transfer (T11) to determine which aspects of the dynamics changed under selection and how. Evolutionary replicates did not vary significantly from each other in any aspect of total cell dynamics, and the small difference in sporulation behaviour among replicates was accounted for by including replicate as a random factor. Therefore, the following results are based on the average of the four replicates (Fig. 2).

The total cell density in our evolved populations at transfer 11 (T11) was 2.75 times higher than the ancestor at transfer 1 (T1) (*P*=7.2×10^−7^). The growth rate from 0 to 6 and 6 to 24 h did not change significantly by T11 (*P*=0.33 and *P*=0.89, respectively). However, the lag time was virtually erased in the evolved populations, from on average 2.12 h at T1 to virtually 0 h at T11 (*P*=4.9×10^−3^, *t*=7.49, df=3). Additionally, total cell density peaked at 24 h at 3.58 times the density of the ancestor, which had an average peak total cell density of 2.37×10^8^ c.f.u. ml^−1^ (*P*=1.3×10^−3^, *t*=−11.69, df=3). Peak total cell density at T11 was followed by a decline in total cell density from 24 to 56 h as compared to a small increase in total cell density in T1 (*P*=4.1×10^−4^). Consequently, total cell density at T11 at 56 h dropped to only 58% of the total cell density of the ancestor at 56 h (4.8×10^8^ to 2.8×10^8^ c.f.u. ml^−1^, *P*=3.9×10^−4^, *t*=17.80, df=3) (Fig. 2a).

The absolute density of endospores at the time of transfer (0 h) decreased significantly by T11 by a factor of 563 on average, from 1.38×10^8^ c.f.u. ml^−1^ at T1 to 2.45×10^5^ c.f.u. ml^−1^ (*P*=1.3×10^−2^). The proportion of endospores (compared to the absolute endospore density at 0 h) germinating from 0 to 6 h increased from 48.5% on average at T1 to 83.7% on average at T11 (*P*=7.0×10^−3^, *t*=−6.64, df=3). However, this did not correspond to a significantly increased rate of germination from 0 to 6 h (*P*=0.12), including when looking at the rate of change of endospore proportion (*P*=0.23).

The rate of endospore formation from 6 to 24 h at T11 was ~2.3 times faster than at the first transfer, which was 0.10, parameterized as log_10_(c.f.u. ml^−1^)/hour (*P*=5.3×10^−5^), and was also 3.8 times faster when considering the rate of change of endospore proportion (*P*=1.4×10^−4^). However, the rate of endospore formation in density and proportion change from 24 to 56 h was not significantly different at T11 (*P*≥0.45). Most dramatically, at the 56 h time point, the proportion of endospores in the population decreased from 84.3% on average at the first transfer to 3.0% on average at the last transfer (*P*=1.3×10^−4^, *t*=25.75, df=3) (Fig. 2b).

### Changes in colony morphology under 56-h nutrient-cycling selection

**Fig. 3.**
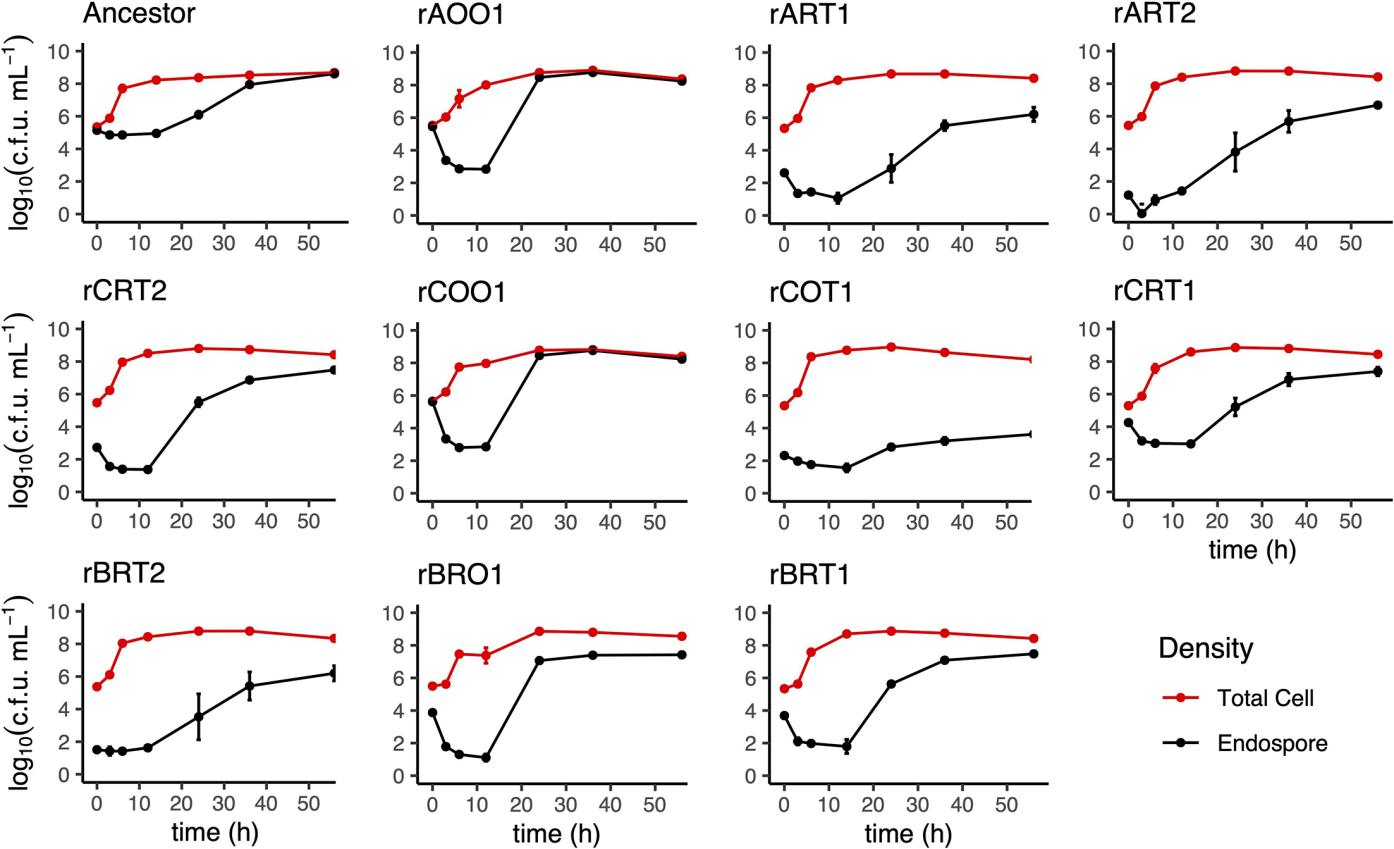
Variability in cell dynamics of isolated evolved strains compared to their ancestor. Ancestor: unevolved original strain of *B. subtilis* 168. Apart from ancestor (upper left), rows from top to bottom correspond to replicates A, C and B. Red dots and lines, total cell density; black dots and lines, endospore density. The second column from the left contains the sporogenous-identified isolates, with the remaining non-ancestor isolates being oligosporogenous (reduced endospore density relative to total cell density). Vertical bars represent sd, which are smaller than the dot if not visible. *N*=3 for all except the Ancestor. *N*=4 for the ancestor.

Colony morphology differences were recorded because, by the fourth transfer, we observed in all replicates a decrease in the relative frequency of opaque brown colonies after incubation on agar for at least 3 days. Some colonies instead were whiter and became translucent the longer they incubated, indicating they had reduced sporulation, either by entirely losing the ability to sporulate (asporogeny) or by reducing the fraction of spores produced (oligosporogeny) (Fig. 1) [1]. The proportion of translucent colonies increased in all replicates by transfer 7, representing on average more than 98.5% of colonies. All seven translucent colonies isolated at the end of the experiment displayed reduced sporulation efficiency, but never complete loss, making them oligosporogenous (Fig. 3).

### Isolate overview

**Fig. 4.**
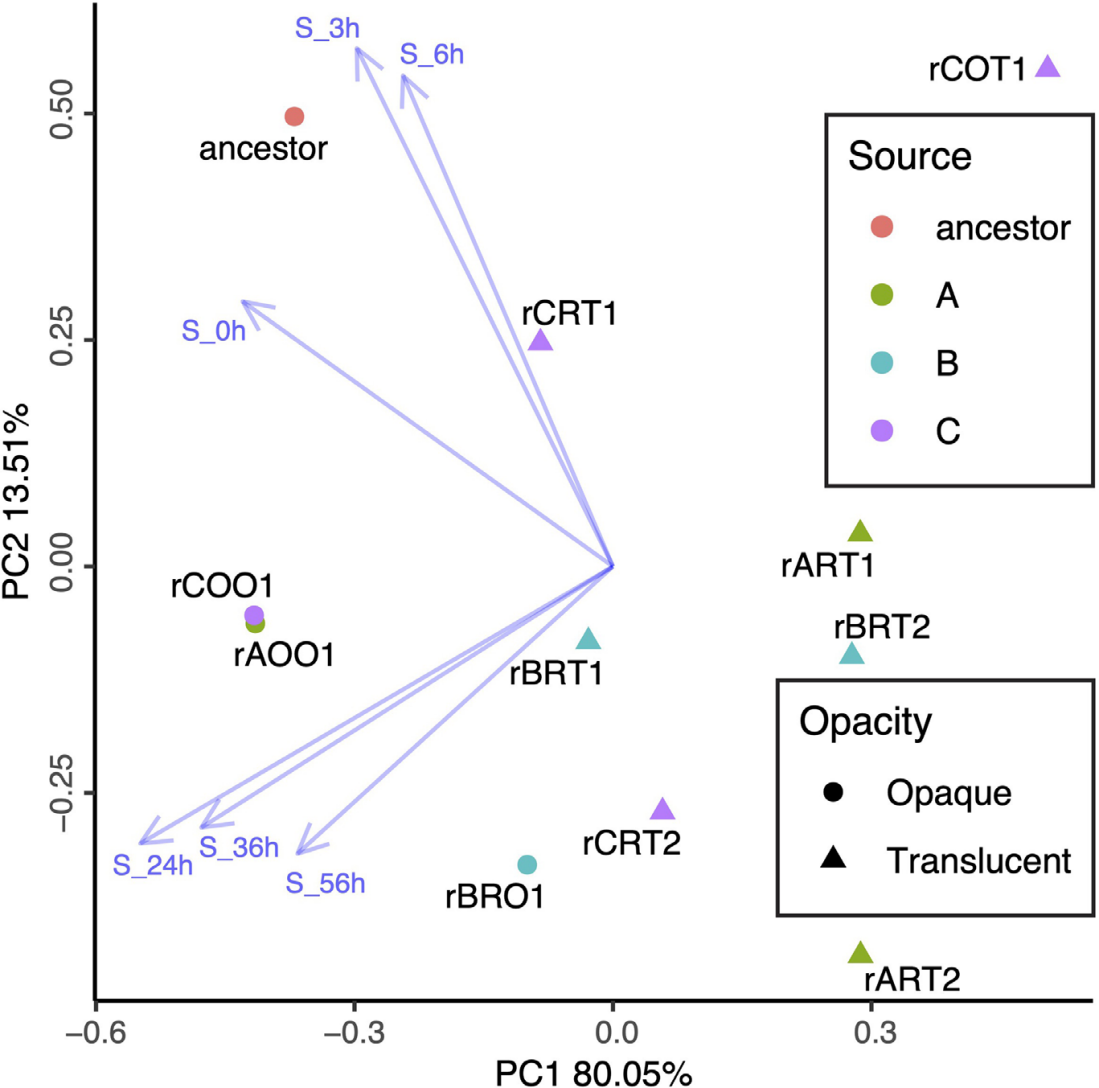
PCA of isolated evolved strains and their common ancestor, showing separate clustering of lower (right) and higher (left) sporulators. Opacity: colony morphology; opaque being sporogenous, translucent being oligosporogenous. Source: the origin of the isolate, being either ancestral (red) or from evolved microcosm A (green), B (cyan) or C (purple). Blue vectors represent the explanatory power of endospore density (S) at time points 0–56 h. Total cell densities were minimally influential and therefore excluded visually from the graph.

Ten evolved isolates were measured for total cell density and endospore density periodically over 56 h of culture in SSM to determine whether a variety of strains existed within each replicate and whether similar strains appeared in parallel across the replicates. When comparing the culture dynamics and colony morphologies of our isolates, it appeared that they could be broadly categorized. Seven of the measured isolates displayed translucent colony morphology, rART1, rART2, rBRT1, rBRT2, rCOT1, rCRT1 and rCRT2, and three isolates displayed opaque brown colony morphology, rAOO1, rBRO1 and rCOO1. Therefore, to validate our visual observations and determine which factors most strongly drove differences among strains, we performed a PCA of our isolates from the selection, together with their ancestor population, using the time series data (Fig. 4). The evolved isolates fell into roughly two clusters separate from the ancestor based on the first two principal components (PC1 and PC2), which together explained 93.56% of the variance. The isolates that exhibited a translucent colony morphology formed one cluster, and those that exhibited an opaque brown colony morphology formed another. Variation in endospore densities explained the differences between isolates more strongly than variation in total cell densities (Fig. 4).

### Behaviour of evolved isolates compared to their ancestor

To further understand the unique aspects of each isolate, we compared each of their time series to the time series of the ancestor via spline regression. At 0 h, all isolates were within ±7.6% of the ancestor’s total cell density of 5.11 log_10_(c.f.u. ml^−1^) (*P*≥0.05). None of the isolates grew significantly faster than the ancestor [0.42 log_10_(c.f.u. ml^−1^)/hour] from 0 to 6 h. However, from 6 to 24 h, rBRO1 and rAOO1 grew significantly faster than the rate of the ancestor [0.05 log_10_(c.f.u. ml^−1^)/hour] (208%, *P*=5.3×10^−4^ and 204%, *P*=7.9×10^−4^, respectively), but rCOO1 did not (*P*=0.12). Interestingly, only rCOT1 and rBRT1 decreased total cell density significantly faster than the ancestor [0.007 log_10_(c.f.u. ml^−1^)/hour] from 24 to 56 h (−397%, *P*=3.3×10^−4^ and −310% *P*=2.9×10^−3^, respectively) (Fig. 3).

All isolates other than rCRT1, rCOO1 and rAOO1 had significantly lower starting endospore density compared to the ancestor (*P*≤1.7×10^−3^), and rCRT1 had near-significant (considering the Bonferroni correction) lower starting endospore density (*P*=0.027). rCOO1, rBRO1, rAOO1 and rBRT1 had significantly faster germination from 0 to 6 h than the ancestor. rCOO1 germinated at 689% (*P*=2.2×10^−6^), rBRO1 at 665% (*P*=5.1×10^−6^), rAOO1 at 653% (*P*=8.0×10^−6^) and rBRT1 at 465% (*P*=3.2×10^−3^) the rate of the ancestor [−0.09 log_10_(c.f.u. ml^−1^)/hour]. Six isolates had significantly faster sporulation from 6 to 24 h than the ancestor. rBRO1 sporulated at 379% (*P*=5.3×10^−14^), rCOO1 sporulated at 376% (*P*=1.0×10^−13^), rAOO1 sporulated at 372% (*P*=1.9×10^−13^), rCRT2 sporulated at 280% (*P*=4.2×10^−7^), rBRT1 sporulated at 254% (*P*=1.5×10^−5^) and rART2 sporulated at 213% (*P*=1.2×10^−3^) the rate of the ancestor [0.10 log_10_(c.f.u. ml^−1^)/hour]. Only rCOO1 and rAOO1 sporulated significantly more slowly than the ancestor from 24 and 56 h. rCOO1 sporulated at 2.4% (*P*=2.1×10^−4^) and rAOO1 sporulated at 2.9% (*P*=2.3×10^−4^) than the rate of the ancestor [0.08 log_10_(c.f.u. ml^−1^)/hour], with rBRO1 also slower and the next closest to significant after the Bonferroni correction (29% and *P*=6.5×10^−3^) (Fig. 3).

### Comparison of behaviour of sporogenous and oligosporogenous isolates

To further understand the differences between the isolates with opaque sporogenous colonies and those with translucent oligosporogenous colonies, we quantified them as groups according to their morphology. The sporogenous isolates displayed efficient germination and retained greater sporulation efficiency (Fig. 4). Sporogenous isolates germinated 99.8% of their endospores on average by 6 h, whereas oligosporogenous isolates germinated 76.4% of their endospores on average in this time (*P*=0.078, *t*=−2.12, df=6). From 6 to 24 h, sporogenous isolates produced 1.11×10^7^ endospores per hour on average, and oligosporogenous isolates produced 8650 endospores per hour on average (*P*=0.17, *t*=−2.12, df=2). By 56 h, the endospore proportion of total cell density for rAOO1 and rCOO1 was 72% on average, whereas rBRO1 had 7% endospores on average, and the translucent isolates were 5% endospores on average (Fig. 3).

### SNPs and indels in evolved isolates

To determine the genetic underpinnings of the observed changes in our evolved populations and isolates, we sequenced the whole genomes of seven of our isolates, representing three of our evolutionary replicate microcosms and covering opaque (rAOO1, rBRO1 and rCOO1) and translucent (rART1, rBRT1, rCOT1 and rCRT2) colony morphologies. Two isolates, rAOO1 and rCOO1, had no notable genome differences from the ancestor but differed in behaviour compared to the ancestor, and not each other (Fig. 3). The SNPs and indels of the remaining five isolates are summarized in Table 1. Four oligosporogenous isolates representing three evolutionary replicates had independent mutations in *kinA*, which encodes a key component of the sporulation phosphorelay. The oligosporogenous isolate without a *kinA* mutation, rCOT1, was also the isolate with the lowest sporulation ability. Other mutations occurred in genes more loosely or peripherally involved in sporulation or germination. Three oligosporogenous isolates had mutations in rRNA genes, including reversions to the published *B. subtilis* 168 sequences in rBRT1. All mutations are listed in Table 1.

## Discussion

This study adds to a body of evidence that the life history strategies of sporulation and germination in endospore-forming bacteria are sophisticated and nuanced bet-hedging strategies evolutionarily as well as physiologically. Endospore-forming bacteria are behaviourally adaptable to nutrient abundance, deficit or in this case something intermediate. Across evolutionary replicates, the conditions of the 56 h nutrient-cycling selection environment yielded parallel outcomes of oligosporogeny, increased germination efficiency and increased peak total cell density followed by a period of starvation. These results are consistent with previous literature showing the evolution of asporogeny or oligosporogeny under similar conditions [7,19, 29]. However, the persistence of strains bearing the unmutated ancestral genome co-occurring with the evolution of oligosporogenous strains is a novel finding. The presence of mutations in genes involved in the phosphorelay, primarily in *kinA*, in oligosporogenous isolates from all tested replicates suggests that the parallel evolution observed at the phenotypic level is partly underlain by parallelism at the genotypic level.

Given that endospore-forming bacteria are thought to be adapted to unpredictable or inconsistent environments, it is interesting that our experimental populations appeared to adjust to the specific length of the period between transfers [24,59]. Behavioural adjustments like the acceleration and increased synchronicity of sporulation might reflect the reliable timing of nutrient renewal and might be an indication of reduced bet-hedging. However, the consistent and predictable nutrient deprivation after 56 h in our culturing conditions appears to warrant some bet-hedging in the form of sporulation, that is, the universal retention of the ability to sporulate with varying degrees of oligosporogeny and particularly the retention of wholly sporogenous strains. The marked decrease in total cell density from 24 to 56 h in our vegetative-dominated evolved populations suggests that a population or mutant lineage composed entirely of asporogenous cells could risk extinction by starvation within that time (Fig. 2a).

Alternatively, the evolution of oligosporogeny might precede the complete loss of sporulation. A more rapid and complete loss of the ability to sporulate might be constrained by stationary phase processes, which rely on regulatory elements shared with sporulation [20]. Stationary phase processes include the toxin/anti-toxin cannibalism pathways and slower growth [20,25, 60, 61]. To avoid disrupting stationary phase processes, a delay in the initiation of sporulation, such as by disrupting the phosphorelay, might accomplish a ‘vegetative enough’ population. If conditions favouring vegetative cells persist, this could provide a basis for further erosion of sporulation ability.

### Drivers of population-level changes in behaviour

Whereas nutrient-rich conditions have led to asporogeny and oligosporogeny in other studies, the study most similar to this one used a growth medium for selection that prohibited sporulation due to the absence of necessary nutrients [42]. The oligosporogeny in our evolved populations is therefore underscored by the fact that SSM is the best known for promoting sporulation in *B. subtilis*. This means that in our study, oligosporogeny was not driven by a nutrient-imposed limitation on the ability to sporulate, but rather occurred in conditions entirely permissive of sporulation [10,41]. Oligosporogeny arose because nutrient renewal in our study was frequent enough to circumvent complete starvation. Without complete starvation, vegetative cells have a growth advantage over spores after transfer owing to the 2 h it takes spores to germinate in SSM [11,14, 62, 63]. The growth advantage of vegetative cells is supported by the virtual absence of a lag time in our evolved populations, which are dominated by vegetative cells (Fig. 2a), a finding consistent with studies in which sporulation ability was lost [11,42].

In addition to oligosporogeny in our evolved populations, we recorded acceleration in sporulation rate from 6 to 24 h in all populations and in six of the ten tested isolates, meaning sporulation is occurring on average earlier and more synchronously. The acceleration in sporulation rate may indicate an investment in endospore quality over quantity, as earlier sporulation forms endospores that germinate earlier and more efficiently [11,14, 42]. This fits particularly well with the opaque colony isolates, all three of which had the most significant acceleration in sporulation and germination.

An investment in endospore quality over quantity is suggested also by the increase in germination proportion in our evolved populations and the increase in germination rate in four of the ten tested isolates. Interestingly, we did not find changes in any genes known to be involved in the initiation or process of germination. However, the conditions of sporulation have a significant effect on the properties of endospores produced, similar to maternal provisioning in plant seeds [15,64]. Therefore, we expect that the changes in germination behaviour can largely be explained by the observed changes in sporulation behaviour. Germination efficiency could also have increased plastically, such as via epigenetic changes, due to the bottleneck of the 1:1,000 dilution at transfer, because dormant endospores would be quickly diluted out [14]. Altogether, it appears that dilution and regular nutrient renewal impose a strict selection against hyperdormancy.

Selection also resulted in much higher peak cell density than the ancestor (Fig. 2a). What might explain this difference is that prior to commitment to sporulation, sporulating cells produce bacteriocins and concurrently become bacteriocin resistant, which results in cannibalization of vegetative cells [25]. It is possible that the dramatically lower density of sporulating cells in our evolved populations means that bacteriocin production is also reduced, which would remove the cannibalism-imposed limit on peak cell density. Alternatively, since sporulation is a resource-intensive process, the lower density of cells undergoing sporulation could simply leave a higher concentration of nutrients for non-sporulating cells, resulting in higher overall cell density.

### Differences in isolate-level changes in behaviour

Isolates differed in behaviour from each other and the ancestor but exhibited parallel evolution, indicating that adaptive divergence occurred within all replicates (Fig. 4). The 56-h nutrient-cycling selection resulted in two distinct phenotypes of isolates, broadly categorized by whether they produce translucent (oligosporogenous) or less frequently, opaque (sporogenous) colonies. The translucent isolates appear to have a ‘vegetative strategy,’ with faster early growth and a much lower sporulation efficiency, so that the majority of cells will be vegetative at the time of transfer to the fresh medium. The opaque isolates appear to have a ‘spore strategy,’ germinating faster and more completely, then sporulating early, quickly, and with greater efficiency, presumably producing high-quality endospores and avoiding competition for nutrients during the stationary phase. The spore strategy might work because it is used by a small proportion of the population, allowing maximum investment in the quality of their endospores. How long and in what proportions the vegetative and spore strategy strains could coexist in this system is something to be explored. The colony dimorphism in this experiment, with consequent ecological divergence, is a phenomenon observed in all evolutionary replicates and therefore another aspect of parallel evolution.

Individual isolates sometimes differed in behaviour from their source populations, indicating that population-level behaviour may be an emergent property of the relationships between evolved strains. For example, the evolved populations significantly lost total cell density from 24 to 56 h, while only two of the ten tested isolates, from different replicates, did the same. Given the variation in our isolates within each evolutionary replicate, and their differing dynamics when isolated, we hypothesize that the evolved populations represent communities of ecologically distinct strains, which may be adapted to the presence of other evolved strains as well as the culture conditions and different niches. Adaptive divergence is a virtually universal phenomenon in extended bacterial cultures beginning with a homogenous ancestor [37,65,71].

### Persistence of the ancestral strain through plasticity

Our finding that the two strongly ‘spore strategy’ isolates had no genetic differences from the ancestral strain indicates that the ancestor’s ability to change plastically, such as through epigenetics, is adaptive enough for it to persist alongside its mutation-bearing descendants. Epigenetic alterations in *B. subtilis* are known to change the timing of sporulation initiation or sensitivity to germinants and could have wider effects as well [14,23]. Whether plasticity alone could continue to carry the ancestral strain through more generations in our experimental conditions is an open question, but the fact that it succeeded for at least 100 generations is notable.

### Genomic changes in isolates

The isolates with varying levels of oligosporogeny had mutations in genes that code for components of the phosphorelay controlling sporulation initiation, all of which are hypothesized to cause a delay in the decision to sporulate. The ‘vegetative strategy’ was commonly derived from mutations in *kinA*, a sporulation-specific histidine kinase. Loss of KinA function inhibits sporulation because it is responsible for increasing phosphorylation of Spo0A, which initiates sporulation commitment [35,72, 73]. However, the isolate rCOT1, which exhibited the largest reduction in sporulation, had a mutation in a well-known sporulation inhibitor, *rapA*. The gene *rapA* encodes an aspartate phosphatase, which dephosphorylates Spo0A, directly counteracting the activity of *kinA*. It is possible that the SNP in isolate rCOT1 in *rapA* (p.Arg105Gln) alters the binding of the RapA inhibitor PhrA, which would result in RapA overly inhibiting the initiation of sporulation [74,76].

The rest of the mutations occurred in genes involved in sporulation but with a less well-documented relationship to it, and no mutations were found in genes not related to sporulation by previous studies. Isolate rCOT1 had a mutation in *infA*, which is peripherally linked to sporulation [77]. The gene *infA* encodes initiation factor IF-1, which interacts with ribosomes and may also be an mRNA chaperone, meaning that its alteration may have a global effect on translation and therefore protein expression [77,78]. In the isolate rART1, the effect of the mutation in *ydfQ* on sporulation or germination is unclear, beyond a small reduction in sporulation proportion when it is deleted, and upregulation of its expression during sporulation [35,79]. The disruption of the *yerC* gene (also called *hisR*) with indels in isolate rART1 is intriguing because the protein it encodes appears to regulate histidine biosynthesis, it is found in mature endospores, its knockout moderately reduces endospore production and it may be upregulated during sporulation [35,79,82].

The mutations in copies of 16S and 23S rRNA are not likely to directly affect sporulation, as changes in their structure generally affect ribosome function, which has global effects on protein translation [83,84]. The identical mutations in *rrnI-16S* in isolates rBRO1 and rCRT2 could suggest an independently derived adaptive change or inheritance of a mutation prior to the separation of the ancestral culture into independent evolutionary replicates. One possibility is that the rRNA mutations create faster growth via more efficient protein translation [84]. However, faster growth is an unlikely consequence of our rRNA mutations, as only rBRO1 grew significantly faster than the ancestor and rCRT2 did not. It is more likely that the mutations represent further adaptation to the presence of kanamycin in our growth medium, making them possible ameliorative mutations [85,86]. Kanamycin targets ribosomes, and 16S rRNA mutation has been documented to cause kanamycin resistance in *Mycobacterium* and even alfalfa [87,89].

In summary, we found a diversity of mutations within the five mutation-bearing isolates, suggesting that this type of selection produced ecologically distinct lineages and is an effective way to explore the genetic bases of evolution, particularly related to sporulation. All oligosporogenous isolates had mutations linked to the phosphorelay controlling sporulation initiation, indicating a high level of parallel evolution. Interestingly, there is indirect evidence of a relationship with sporulation for each of the non-phosphorelay mutations [35,77]. Therefore, it appears that reducing the functionality of the sporulation phosphorelay was advantageous in our selection conditions, and the sporulation roles of the non-phosphorelay mutations are worth further investigation.

### Future directions

The issue of starvation appears to be central to the maintenance of sporulation ability. In a separate study, *B. subtilis* sporulation efficiency was not increased through culling of vegetative cells via heat prior to a starvation period [10]. However, higher sporulation efficiency was achieved by passaging *Bacillus atrophaeus* every 12–18 months, imposing an extremely extended period of starvation [18]. In the present study, the retention of sporulation ability despite reductions in sporulation efficiency and the possible presence of two distinct sporulation strategies strongly suggest an adaptation to the specific length of our culture period between transfers, particularly related to the starvation period. We are testing this hypothesis by repeating the selection process with longer and shorter nutrient cycles, with the longer period encompassing complete starvation and the shorter period closer to continuous nutrient availability. We hope these studies will contextualize the dramatic changes we found in virtually every aspect of the behaviours resulting from our 56-h nutrient-cycling selection.

## Abbreviations

LB: lysogeny broth
PCA: principal component analysis
SSM: Schaeffer’s sporulation medium
